# Case Report: A family with X-linked ichthyosis identified by secondary findings of non-invasive prenatal testing

**DOI:** 10.3389/fmed.2026.1740641

**Published:** 2026-03-12

**Authors:** Shuzhen Li, Fuxin Chen, Lihua Li, Miaolian Zhang, Huahua Li, Suihua Feng, Xiaohong Ruan, Qiang Zhao

**Affiliations:** 1 Reproductive Medicine Center, Jiangmen Central Hospital, Jiangmen, China; 2 Department of Obstetrics and Gynecology, Jiangmen Central Hospital, Jiangmen, China; 3 Clinical Transformation and Application Key Lab for Obstetrics and Gynecology, Pediatrics, and Reproductive Medicine of Jiangmen, Jiangmen, Guangdong, China

**Keywords:** chromosome microarray analysis, copy number variation, NiPt, prenatal screening, X-linked ichthyosis

## Abstract

We report a family with X-linked ichthyosis (XLI), in which the non-invasive prenatal testing (NIPT) results from the pregnant woman revealed a deletion of approximately 2 Mb at Xp22.31, confirmed by chromosome microarray analysis. This deletion includes the *steroid sulfatase (STS)* gene, which is responsible for XLI, and clinical features of XLI were also found in family members. XLI is a recessive hereditary skin disease characterized by deep brown polygonal scales, and its clinical manifestations are not obvious, making genetic diagnosis difficult for patients. The incidental findings from this NIPT suggest that copy number variations (CNVs) detected by NIPT can help predict pathogenic CNVs in the fetus and even in the entire family genome. We should pay more attention to CNVs identified by NIPT during prenatal screening.

## 1 Introduction

Since Professor Dennis Lo discovered fetal cell-free DNA in maternal peripheral plasma in 1997, non-invasive prenatal testing (NIPT) technology has been widely used in prenatal screening for pregnant women (1–3). NIPT provides “non-invasive” prenatal testing on fetal cell-free DNA in maternal peripheral blood using high-throughput sequencing technology, which can screen for fetal chromosomal aneuploidies and detect copy number variations (CNVs) in the fetal genome, specifically increases or decreases in the number of copies of large DNA sequences (typically longer than 1,000 base pairs) on the genome. Although NIPT technology still faces challenges in accurately diagnosing fetal genomic CNVs, pathogenic CNVs suggested by NIPT results are still clinically significant.

**Figure 1:**
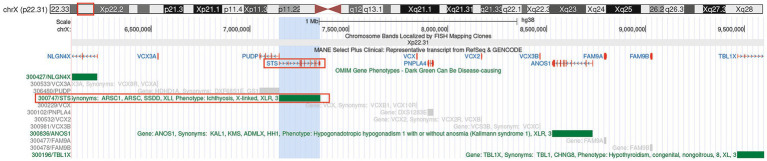
Genome annotation map of the Xp22.31 region. Gene annotation map was generated using the UCSC Genome Browser (hg38 assembly) https://genome.ucsc.edu/s/cherish/Xp22.31.

X-linked ichthyosis (XLI, OMIM # 308100) is an X-linked recessive hereditary skin disease caused by a deficiency of steroid sulfatase (4; Figure 1). The incidence in males is approximately 1 in 6,000 (5). Affected males typically exhibit widely distributed polygonal, translucent scales shortly after birth, which are gradually replaced by larger, darker brown-gray scales localized to the neck, trunk, lower extremities, and extensor surfaces (6). Nearly all cases of XLI demonstrate a specific corneal dystrophy, appearing as a thin, gray-white “frosted” layer deep in the stroma, a finding that distinguishes XLI from other forms of ichthyosis (7). Additionally, approximately 10–15% of patients with XLI present with cryptorchidism (6). This hereditary disease not only significantly affects the patient’s appearance but may also be accompanied by a range of health issues, such as mental health problems, as evidenced by higher levels of mood disorder diagnoses and symptoms (8). Studies have shown that *steroid sulfatase (STS)* gene deletions, which are the cause of XLI, can lead to attention deficit hyperactivity disorder (ADHD) with predominantly inattentive symptoms (9). Furthermore, when the deletion extends to include neighboring genes such as *neuroligin 4 (NLGN4)* and *short stature homeobox (SHOX)*, it may also result in autism and intellectual disability (9, 10). Currently, the diagnosis of XLI mainly relies on clinical manifestations and genetic testing, but early diagnosis and intervention are still insufficient, and the risk of misdiagnosis remains. Early genetic screening can effectively reduce the incidence of hereditary diseases and provide families with opportunities for early intervention.

The pathogenic gene for XLI is the *STS* gene located at Xp22.31, which contains 10 exons and spans approximately 146 kb. *STS* is a membrane-bound microsomal enzyme that acts as a metabolic precursor for estrogens, androgens, and cholesterol (11–13). In 85–90% of XLI cases, it is caused by deletion CNVs involving the *STS* gene, with only a few cases resulting from small insertions, deletions, or point mutations (14). Due to the non-obvious impact of XLI clinical symptoms on the patient’s life, patients rarely receive clinical and genetic diagnoses. In this article, we report a family with XLI identified through incidental findings from NIPT.

## 2 Materials and methods

This study was approved by the Ethics Committee of Jiangmen Central Hospital and conducted as per the Declaration of Helsinki. All participants provided written informed consent after being fully briefed on the study. For all diagnostic and treatment activities for the fetus, the pregnant woman is informed and signs an informed consent form.

Venous blood (5 mL) was collected from each subject using EDTA-K anticoagulant tubes and centrifuged for 10 min at 1,600×*g* at 4 °C within 8 h after blood collection to obtain cell-free plasma. Plasma circulating cell-free DNA (cfDNA) was extracted from maternal plasma using the Circulating Nucleic Acid kit (Berry Genomics, Beijing, China). DNA library was constructed using enzymatic reactions, molecular labeling, and PCR. DNA fragments were subjected to end repair and linker ligation. After PCR amplification and pooling, single-strand cyclization and DNA nanosphere preparation were carried out to construct a library for sequencing. Each sample was sequenced using the BGISEQ-500 platform and a combinatorial probe-anchored polymer sequencing method, and bioinformatics analysis was performed using BGI Halos software (Shenzhen, China).

Fetal DNA was extracted from amniotic fluid samples, and DNA from other family members was extracted from peripheral blood. These DNA samples underwent chromosome microarray analysis (CMA) (750 k, Affymetrix, Santa Clara, USA).

Ultrasound, fundus examination, and sex hormone quantification were used as auxiliary examinations.

## 3 Results

**Figure 2:**
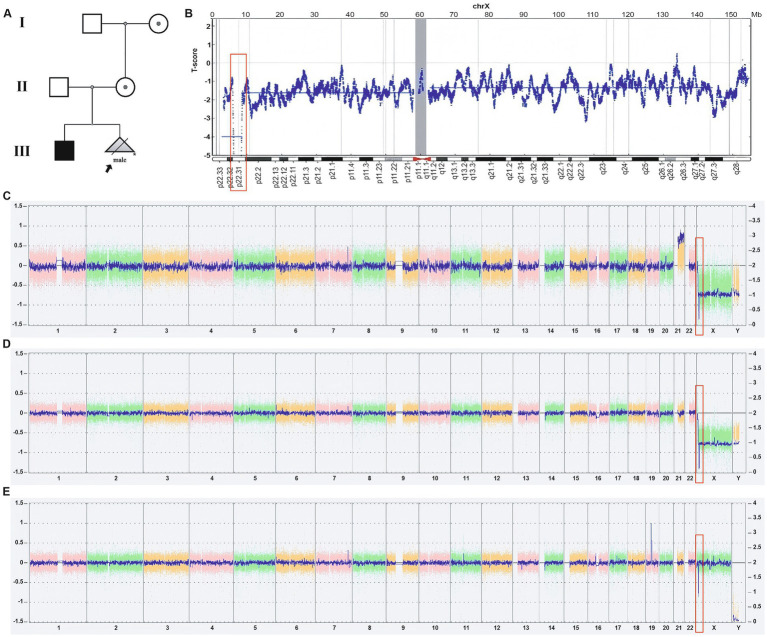
Pedigree analysis of the proband (indicated by black arrows) and the results of NIPT and CMA. **(A)** The proband’s mother and grandmother were confirmed to be carriers of the deletion mutation by CMA testing. The proband’s elder brother has been diagnosed with XIL. **(B)** The NIPT result of the pregnant woman showed a high risk of an approximately 2 Mb deletion at Xp22.31, which also indicated a high risk for trisomy 21 (not shown in the figure). **(C)** CMA results from the pregnant woman’s amniotic fluid showed the fetus’s karyotype was 47, XY, +21, and a 1.68 Mb deletion of Xp22.31. **(D,E)** The CMA test results of the proband’s elder brother **(D)** and mother **(E)** showed that both have a deletion of approximately 1.68 Mb at Xp22.31.

A 35-year-old pregnant woman underwent a routine ultrasound examination at Jiangmen Central Hospital at 12 weeks’ gestation. The results showed that the measurement of fetal nuchal translucency (NT) was 3.7 mm and revealed the absence of the fetal nasal bone. Meanwhile, at 14 weeks and 1 day of gestation, she underwent NIPT. The results indicated a high risk for trisomy 21 and a hemizygous deletion of approximately 2 Mb at Xp22.31 (Figures 2A,B). The pregnant woman then received genetic counseling and underwent amniocentesis at 15 weeks and 6 days of gestation for CMA, to determine if the fetus had trisomy 21. The CMA results showed the fetal karyotype as 47, XY, +21, along with a homozygous deletion at Xp22.31 (6455151-8141076), approximately 1.68 Mb in size (Figure 2C). This deletion involves *STS*, *VCX3A*, *HDHD1*, *VCX*, and *PNPLA4*, and searching CNV-related disease databases (such as Decipher, ISCA) suggests that this deletion may lead to XLI.

**Figure 3:**
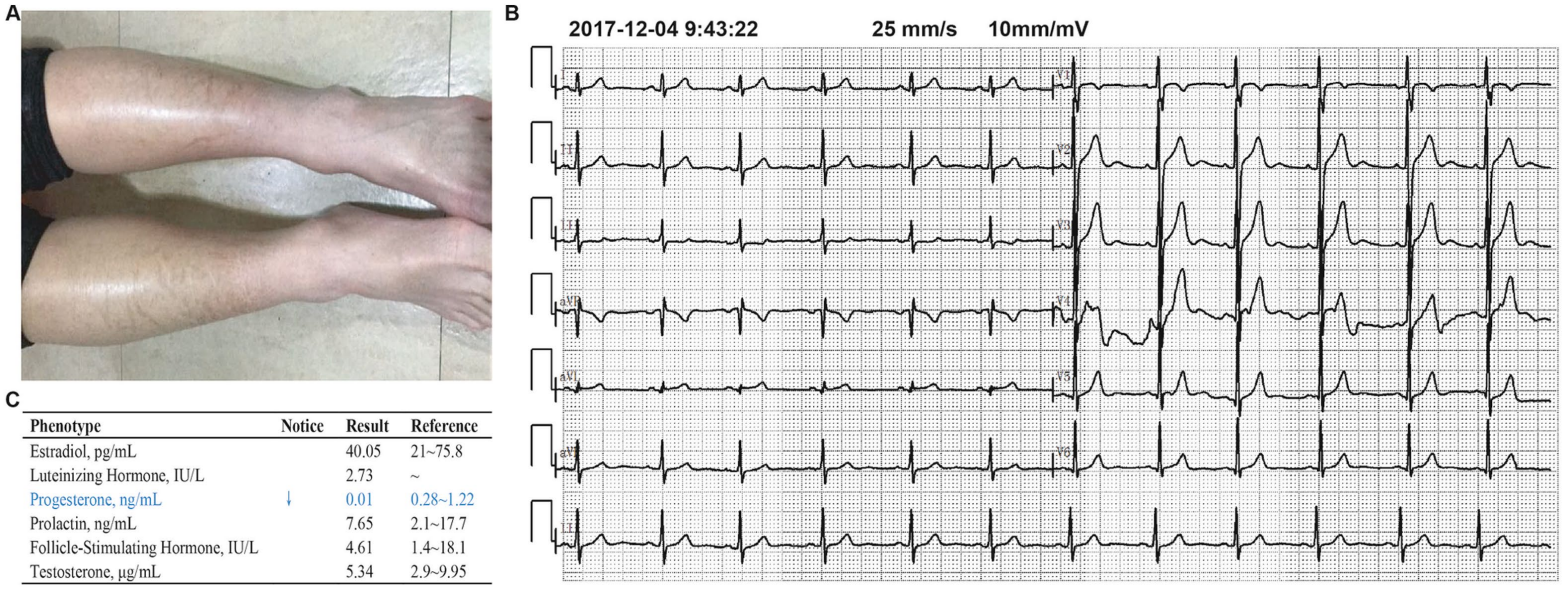
Examination results for the proband’s elder brother (XLI patient). **(A)** The patient’s skin was covered with dark brown polygonal scales. **(B)** The electrocardiogram (ECG) examination results of the patient showed no abnormalities. **(C)** The test results of the patient’s sex hormones showed no abnormalities.

The pregnant woman’s reproductive history is G2P1, and she has a 14-year-old son whose upper and lower limbs are covered with dry, rough, thickened, and dark scales (Figure 3A), while the scalp, neck, and trunk are unaffected. These symptoms have been present since childhood. The boy does not exhibit corneal opacity or cryptorchidism. Electrocardiogram and sex hormone results (estradiol, luteinizing hormone, progesterone, follicle-stimulating hormone, and testosterone) are also normal (Figures 3B,C). During clinical consultation, it was noted that he is easily angered and irritable (15), even shouting at the clinic. However, as he refused to be examined by a psychiatrist, we could not determine if he had ADHD. The boy’s CMA results show he has a hemizygous deletion at Xp22.31, while his mother’s CMA results indicate she is a heterozygous carrier of the deletion (arrXp22.31(6449836-8135568)x1), without any other phenotypic manifestations (Figures 2D,E). No other family members exhibit symptoms of XLI.

## 4 Discussion

We identified a family carrying a 1.68 Mb deletion at Xp22.31, with a 14-year-old boy exhibiting XLI phenotype, consistent with his genetic diagnosis results. This finding originated from incidental findings in the NIPT results of the pregnant women in this family. This study’s innovation lies in offering an effective strategy for the early diagnosis of CNV syndromes similar to XLI through NIPT, thereby providing more precise support for families with high-risk pregnancies (16–18).

The XLI involved in this study is not a life-threatening or severely disabling disease; therefore, it should be discussed through medical ethics and adequate genetic counseling, giving the family the choice to pursue prenatal or pre-implantation genetic diagnosis for intervention related to this condition. This case also suggests that in clinical diagnosis and treatment, we should pay more attention to CNVs indicated by NIPT. Although based on our internal data and published data, the positive predictive value of NIPT for CNVs is only approximately 30%, when CNVs involve known microdeletion/microduplication syndromes, we should evaluate their impact on quality of life and ask about family history. If necessary, we will also conduct prenatal diagnosis to exclude microdeletion/microduplication syndromes with severe clinical phenotypes.

## Data Availability

The raw data supporting the conclusions of this article will be made available by the authors, without undue reservation.
